# Using adaptive magnetic resonance image‐guided radiation therapy for treatment of inoperable pancreatic cancer

**DOI:** 10.1002/cam4.2100

**Published:** 2019-04-01

**Authors:** Soumon Rudra, Naomi Jiang, Stephen A. Rosenberg, Jeffrey R. Olsen, Michael C. Roach, Leping Wan, Lorraine Portelance, Eric A. Mellon, Anna Bruynzeel, Frank Lagerwaard, Michael F. Bassetti, Parag J. Parikh, Percy P. Lee

**Affiliations:** ^1^ Department of Radiation Oncology Washington University School of Medicine St. Louis Missouri; ^2^ Department of Radiation Oncology David Geffen School of Medicine at UCLA Los Angeles California; ^3^ Department of Human Oncology University of Wisconsin School of Medicine and Public Health, Carbone Cancer Center Madison Wisconsin; ^4^ Department of Radiation Oncology Sylvester Comprehensive Cancer Center, University of Miami Miami Florida; ^5^ Department of Radiation Oncology VU University Medical Center Amsterdam Netherlands; ^6^ Present address: Department of Radiation Oncology Moffitt Cancer Center 12902 USF Magnolia Drive Tampa Florida 33612; ^7^ Present address: Department of Radiation Oncology University of Colorado 1665 Aurora Court, Suite 1032 Aurora Colorado 80045; ^8^ Present address: Department of Radiation Oncology Henry Ford Cancer Institute 2799 West Grand Blvd Detroit Michigan 48202

**Keywords:** magnetic resonance imaging, pancreatic cancer, radiation therapy

## Abstract

**Background:**

Adaptive magnetic resonance imaging‐guided radiation therapy (MRgRT) can escalate dose to tumors while minimizing dose to normal tissue. We evaluated outcomes of inoperable pancreatic cancer patients treated using MRgRT with and without dose escalation.

**Methods:**

We reviewed 44 patients with inoperable pancreatic cancer treated with MRgRT. Treatments included conventional fractionation, hypofractionation, and stereotactic body radiation therapy. Patients were stratified into high‐dose (biologically effective dose [BED_10_] >70) and standard‐dose groups (BED_10_ ≤70). Overall survival (OS), freedom from local failure (FFLF) and freedom from distant failure (FFDF) were evaluated using Kaplan‐Meier method. Cox regression was performed to identify predictors of OS. Acute gastrointestinal (GI) toxicity was assessed for 6 weeks after completion of RT.

**Results:**

Median follow‐up was 17 months. High‐dose patients (n = 24, 55%) had statistically significant improvement in 2‐year OS (49% vs 30%, *P* = 0.03) and trended towards significance for 2‐year FFLF (77% vs 57%, *P* = 0.15) compared to standard‐dose patients (n = 20, 45%). FFDF at 18 months in high‐dose vs standard‐dose groups was 24% vs 48%, respectively (*P* = 0.92). High‐dose radiation (HR: 0.44; 95% confidence interval [CI]: 0.21‐0.94; *P* = 0.03) and duration of induction chemotherapy (HR: 0.84; 95% CI: 0.72‐0.98; *P* = 0.03) were significantly correlated with OS on univariate analysis but neither factor was independently predictive on multivariate analysis. Grade 3+ GI toxicity occurred in three patients in the standard‐dose group and did not occur in the high‐dose group.

**Conclusions:**

Patients treated with dose‐escalated MRgRT demonstrated improved OS. Prospective evaluation of high‐dose RT regimens with standardized treatment parameters in inoperable pancreatic cancer patients is warranted.

## 1 INTRODUCTION

Radiation therapy (RT) is controversial in the treatment of inoperable pancreatic cancer. The results of the phase III randomized LAP07 trial illustrate that conventional doses of 3D conformal radiation (3DCRT) do not confer an overall survival (OS) advantage over chemotherapy alone in locally advanced pancreatic cancer (LAPC).^1^ Investigators have evaluated RT techniques other than 3DCRT to improve outcomes. Retrospective and phase I/II studies of stereotactic body radiation therapy (SBRT) have shown local control outcomes generally exceeding 80% but no change in OS as compared to historical data and some concern with unfavorable toxicity.^2^, ^3^, ^4^, ^5^, ^6^, ^7^, ^8^ Multiple institutions have demonstrated favorable survival results with intensity‐modulated radiation therapy (IMRT) after induction chemotherapy.^9^, ^10^ Krishnan et al have demonstrated that dose escalation using simultaneous integrated boost (SIB) with IMRT improved survival and local control in LAPC compared to standard radiotherapy doses in a single‐institution retrospective study.^11^ These techniques suggest opportunities to potentially improve outcomes with dose escalation with RT.

Adaptive magnetic resonance imaging (MRI)‐guided radiation therapy (MRgRT) is a novel modality that potentially allows for dose escalation while minimizing excessive radiation dose to the organs at risk (OAR). MRgRT offers excellent visualization of the stomach, duodenum, small, and large intestines in the setting of abdominal RT and thus can account for interfractional variability of these organs.^12^, ^13^ The ability to adapt treatment plans daily allows for improved target volume coverage while meeting OAR constraints that is necessitated by daily gastrointestinal (GI) organ motion and deformation.^14^, ^15^, ^16^ While dose escalation with MRgRT appears technically feasible and can spare OARs from high doses of radiation, it is not clear whether this leads to changes in clinical outcomes. This multi‐institutional study evaluated clinical outcomes of inoperable pancreatic cancer patients treated with MRgRT and compared patients treated with and without adaptive dose‐escalated regimens.

## 2 METHODS

This study was a multi‐institutional, retrospective, cohort study based on data from 5 institutions. Eligible patients had biopsy‐proven, inoperable, pancreatic cancer treated with MRgRT from 2014 through 2016. All patients were evaluated with diagnostic computed tomography (CT) imaging or MRI. Patients deemed medically inoperable were included in our study as well. The study excluded patients with prior pancreas‐directed RT, pancreatic surgery or any clinical‐radiographic evidence of distant metastasis prior to initiation of RT. Research conformed to the Helsinki Declaration and satisfied retrospective review requirements for each institution.

Systemic therapy was determined by the local medical oncologist or institutional study protocol. Induction chemotherapy regimens included nab‐paclitaxel plus gemcitabine, FOLFIRINOX, gemcitabine alone, FOLFOX, and combined regimens. Concurrent chemotherapy was delivered on the day of radiation treatments with conventional fractionated radiation treatments and hypofractionated radiation treatments. Concurrent regimens consisted of nab‐paclitaxel plus gemcitabine, gemcitabine alone, and capecitabine alone. After RT, patients received systemic therapy according to institutional preferences.

MRgRT (ViewRay MRIdian System, Oakwood Village, OH) was used to treat all patients included in this study, but simulation and planning techniques varied between institutions. The Supplemental methods and Table S1 offer a detailed overview of simulation and planning information for each institution. In general, patients were simulated with a planning CT and planning 0.35T MRI scan in the supine position. Gross tumor volume (GTV) included the primary tumor with or without inclusion of any enlarged regional lymph nodes (>1 cm in short axis) and was delineated using MRI simulation, CT simulation and diagnostic imaging. Some institutions contoured regional lymph nodes into the clinical target volume (CTV) while others did not treat enlarged regional lymph nodes and considered GTV equal to CTV. Planning target volume (PTV) was generated with a 5 mm isotropic margin from GTV or CTV.

Adaptive MRgRT treatments were delivered for patients receiving 15 or fewer fractions and required patients to receive a volumetric MRI prior to each fraction. Patients were aligned daily to the GTV based on their volumetric MRI, and their radiation plan was evaluated on their current anatomy. If the plan did not meet OAR constraints or the target was not covered adequately, a new plan was created using the same beam angles. If the new plan did not resolve constraint violations and/or improve coverage, then the prior plan was utilized for treatment of that fraction. Adaptive MRgRT plans prioritized strict OAR constraints even at the expense of PTV coverage. However, if favorable OAR anatomy was noted during adaptive treatment, dose escalation to improve PTV coverage could be performed while maintaining OAR constraints.^14^, ^15^, ^16^, ^17^ In some cases, an adaptive plan could be generated to treat above the prescription dose. For patients receiving >15 fractions of treatment, nonadaptive MRgRT was delivered using conventional treatment planning with no adjustment for daily internal anatomy.

In order to compare the effects of various dose and fractionation schedules on outcomes, the biologically effective dose (BED_10_) was calculated for each patient using the radiation prescription dose and alpha/beta ratio of 10 as previously reported in the literature.^11^ Patients receiving a radiation prescription with BED_10_ >70 Gy were grouped in the high‐dose cohort while those receiving a radiation prescription with BED_10_ ≤70 Gy were grouped in the standard‐dose cohort. Table 1 provides complete RT regimen details for the study patients.

**Table 1:** Radiation therapy regimens

RT technique	Prescription dose & fractionation	Number of patients	Median BED_10_[range]	Minimum PTV dose range	Mean PTV dose range	Maximum PTV dose range
Conventionally fractionated	40‐55 Gy in 25‐28 fractions	13	55.5 [38.2‐67.1]	36.9‐49.0 Gy	42.4‐57.1 Gy	45.0‐61.6 Gy
Conventional SBRT	30‐35 Gy in 5 fractions	6	55.8 [48.0‐59.5]	23.6‐29.9 Gy	31.2‐38.3 Gy	35.6‐45.1 Gy
High‐dose SBRT	40‐52 Gy in 5 fractions	16	77.6 [72.0‐106.1]	13.3‐37.8 Gy	38.1‐60.6 Gy	44.6‐68.1 Gy
Hypofractionated	50‐67.5 Gy in 10‐15 fractions	9	82.7 [67.8‐97.9]	10.8‐37.8 Gy	45.5‐66.3 Gy	60.3‐88.1 Gy

BED_10_, biologically effective dose; PTV, planning target volume; RT, radiation therapy; SBRT, stereotactic body radiation therapy.

### 2.1 Outcomes and statistical analysis

Endpoints included OS, freedom from local failure (FFLF), freedom from distant failure (FFDF) and rate of acute grade 3 or higher GI toxicities. All time to endpoint calculations were performed from start date of RT. Surviving patients were censored at last follow‐up. Both local and distant failure events were determined using routine clinical‐radiographic studies. Local failure was defined as radiographic progression noted at the primary tumor site or regional nodes. Distant failure was defined as radiographic progression at any other site. Kaplan‐Meier curves were generated and differences between groups were determined using the log‐rank method. Acute GI toxicity was graded based on the Common Terminology Criteria for Adverse Events version 4 and recorded from start of RT until 6 weeks after completion of RT.

Baseline patient characteristics were evaluated by Fisher's exact testing for categorical variables and Mann‐Whitney *U* testing for continuous variables. Statistical significance was defined as a *P* < 0.05 and all testing was two‐sided. Univariate (UVA) and multivariate (MVA) analyses were performed for OS using Cox regression. CA 19‐9 was analyzed as a binary categorical variable (cutoff value of 37 U/mL based on prior studies^18^). As the number of events was limited, only factors with univariate *P* < 0.05 were included in the MVA. The MVA defined significance as a *P* < 0.05. Statistical analyses were performed using IBM SPSS Statistics, version 23 (IBM Corp., Armonk, NY) and SAS (Version 9.4, SAS Institute Inc, Cary, NC).

## 3 RESULTS

A total of 44 patients were included in the study (high‐dose, n = 24; standard‐dose, n = 20). Concurrent chemotherapy was administered with all the conventional fractionated patients and all but two hypofractionated radiation patients. As expected, treatment fraction adaptation was more common in the high‐dose group (83%) vs the standard‐dose group (15%). After completion of RT, six patients (two in high‐dose group and four in the standard‐dose group) underwent surgical resection with five patients (two in high‐dose, three in standard‐dose) demonstrating microscopically negative margins. Further patient and treatment characteristics are listed in Table 2.

**Table 2:** Patient, tumor and treatment characteristics

Characteristics	Overall (N = 44)	High‐dose (N = 24)	Standard‐dose (N = 20)	*P*‐value
Age at diagnosis (y) (median [range])	66 [47‐85]	68 [51‐85]	61 [47‐84]	<0.01
ECOG PS (%)				
0	13 (29.5)	10 (41.7)	3 (15.0)	0.11
1	29 (65.9)	13 (54.2)	16 (80.0)	
2	2 (4.6)	1 (4.1)	1 (5.0)	
Sex				
Male	26 (59.1)	14 (58.3)	12 (60.0)	1.00
Female	18 (40.9)	10 (41.7)	8 (40.0)	
Maximum tumor dimension at diagnosis (cm)^a^	N = 41 3.5 [1.5‐6.7]	N = 22 3.6 [1.5‐6.7]	N = 19 3.1 [2.0‐6.5]	0.73
Tumor location				
Head	30 (68.2)	17 (70.8)	13 (65.0)	0.75
Body/tail	14 (31.8)	7 (29.2)	7 (35.0)	
CA 19‐9 at diagnosis (U/mL)^a^	N = 40 119.5 [0.8‐5645.0]	N = 21 313.0 [17.0‐5645.0]	N = 19 63.0 [0.8‐2350.0]	0.01
Stage				
BRPC	10 (22.7)	4 (16.7)	6 (30.0)	0.41
LAPC	32 (72.7)	18 (75.0)	14 (70.0)	
Medically inoperable	2 (4.6)	2 (8.3)	0	
Adjacent organ invasion	4 (9.1)	3 (12.5)	1 (5.0)	0.61
Node positive disease	9 (20.5)	4 (16.7)	5 (25.0)	0.71
Post‐RT pancreatectomy	6 (13.6)	2 (8.3)	4 (20.0)	0.39
Planning tumor volume (cc)	87.6 [13.8‐426.0]	73.3 [13.8‐239.0]	123.5 [31.0‐426.0]	0.03
Number of fractions adapted per patient	2.5 [0‐15]	5 [0‐15]	0 [0‐13]	<0.01
Induction chemotherapy				
Nab‐paclitaxel and gemcitabine	16 (36.3)	8 (33.3)	8 (40.0)	0.29
Gemcitabine alone	1 (2.3)	0	1 (5.0)	
FOLFIRINOX	19 (43.2)	9 (37.5)	10 (50.0)	
FOLFOX	1 (2.3)	1 (4.2)	0	
Multiple regimens	4 (9.1)	4 (16.7)	0	
None	3 (6.8)	2 (8.3)	1 (5.0)	
Duration of induction chemotherapy (mo)	3.0 [0‐11.5]	3.9 [0‐11.5]	1.7 [0‐7.4]	0.19
Concurrent chemotherapy				
Nab‐paclitaxel and gemcitabine	9 (20.5)	3 (12.5)	6 (30.0)	0.01
Gemcitabine alone	4 (9.1)	0	4 (20.0)	
Capecitabine	7 (15.9)	3 (12.5)	4 (20.0)	
None	24 (54.5)	18 (75.0)	6 (30.0)	
Post‐RT chemotherapy (maintenance and/or salvage)	30 (68.2)	17 (70.8)	13 (65.0)	0.75

BRPC, borderline resectable pancreatic cancer; ECOG PS, Eastern Cooperative Oncology Group performance status; FOLFIRINOX, fluorouracil, leucovorin, irinotecan, oxaliplatin; FOLFOX, fluorouracil, leucovorin, oxaliplatin; LAPC, locally advanced pancreatic cancer; RT, radiation therapy.

a Data not available for all patients.

**Figure 1:**
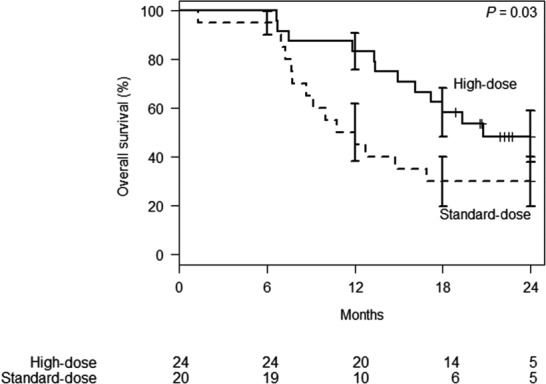
Overall survival from start of radiation therapy stratified by biologically effective dose (BED_10_). Standard error bars displayed at each 6‐mo timepoint

**Figure 2:**
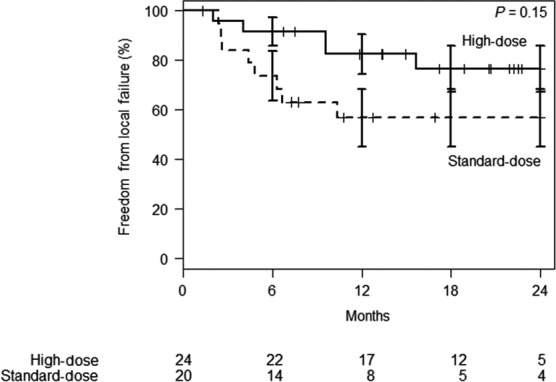
Freedom from local failure from start of radiation therapy stratified by biologically effective dose (BED_10_). Standard error bars displayed at each 6‐mo timepoint

Median follow‐up was 17 months (24 months for surviving patients). OS at 2 years from start of radiation in the high‐dose vs standard‐dose groups was 49% (95% confidence interval [CI]: 28‐69%) vs 30% (95% CI: 10‐50%), respectively (*P* = 0.03, Figure 1). FFLF at 2 years from radiation was 77% (95% CI: 58‐95%) for the high‐dose group vs 57% (95% CI: 34‐80%, *P* = 0.15, Figure 2) for the standard‐dose group. FFDF at 18 months was not significantly different between the high‐dose and standard‐dose groups [24% (95% CI: 5‐44%) vs 48% (95% CI: 23‐73%), respectively, *P* = 0.92, Figure S1]. The OS calculated from diagnosis had similar differences between the groups, with 67% survival at 2 years in the high‐dose group and 30% survival at 2 years in the standard‐dose group (*P* = 0.03). On MVA, neither high‐dose RT group nor length of induction chemotherapy was independently predictive of OS (Table 3). Due to a number of patients receiving the same radiation prescription, an exploratory analysis was performed to evaluate OS from diagnosis stratified by BED_10_ of maximum point dose in target volume (MaxBED_10_); a swimmer's plot was generated to illustrate the findings (Figure S2).

**Table 3:** OS univariate and multivariate cox regression analysis

Characteristic	Univariate HR [95% CI]	*P*‐value	Multivariate HR [95% CI]	*P*‐value
Older age^a^	1.01 [0.96‐1.05]	0.84		
ECOG PS >0	1.05 [0.46‐2.39]	0.90		
Male sex	1.01 [0.48‐2.15]	0.97		
Larger tumor size^a^	0.94 [0.68‐1.29]	0.68		
Tumor located in head	0.64 [0.30‐1.40]	0.27		
CA 19‐9 >37 U/mL	2.75 [0.82‐9.17]	0.10		
BRPC	1.15 [0.49‐2.70]	0.76		
Adjacent organ invasion	0.57 [0.14‐2.43]	0.45		
Node positive disease	1.28 [0.50‐3.25]	0.61		
Pancreatectomy	0.32 [0.08‐1.35]	0.12		
Larger PTV^a^	1.00 [1.00‐1.01]	0.56		
High‐dose RT	0.44 [0.21‐0.94]	0.03	0.56 [0.25‐1.26]	0.16
Longer duration of induction chemotherapy^a^	0.84 [0.72‐0.98]	0.03	0.87 [0.74‐1.03]	0.11
Concurrent chemotherapy	1.03 [0.49‐2.17]	0.94		
Post‐RT chemotherapy	0.86 [0.39‐1.92]	0.72		

BRPC, borderline resectable pancreatic cancer; CI, confidence interval; ECOG PS, Eastern Cooperative Oncology Group performance status; HR, hazard ratio; OS, overall survival; PTV, planning tumor volume; RT, radiation therapy.

a Characteristic analyzed as a continuous variable in regression analysis.

Severe acute GI toxicity (grade 3 or higher) occurred in three patients (7%). All three patients were in the standard‐dose cohort and received concurrent gemcitabine‐based chemotherapy. Two patients developed abdominal infections requiring hospitalization, and one patient experienced a grade 4 duodenal ulcer requiring intensive care unit admission. No patient died of their acute toxicity.

## 4 DISCUSSION

This study suggests that adaptive MRgRT is a promising approach to advance the treatment of inoperable pancreatic cancer. MaxBED_10_ of treatment was shown in our study to be associated with improved OS and FFLF. By using online adaptive treatment planning to spare OARs and optimize RT dose delivery to target volumes, MRgRT appears to be a safe technique for ablative dose escalation.

Dose escalation in pancreatic cancer is an area of keen interest in the radiation oncology community. Early approaches included intraoperative radiation therapy (IORT) where visible tumor was irradiated with a single electron portal. This procedure was limited to those fit for surgical exploration with constraints on size of tumors. Modern reports of this technique show median OS of 25 months from diagnosis for patients receiving IORT without resection.^19^ With the advent of 3DCRT and IMRT approaches, moderate dose escalation to BED from 60‐70 Gy became possible. A recent review of 134 patients by Huguet et al noted a median survival from diagnosis of 23 months for patients treated with 56 Gy/28 fractions (BED_10_ = 67.2 Gy) with chemoradiation.^9^

Stereotactic body radiation therapy led to attempts at dose escalation but with a concomitant increase in toxicity. Schellenberg et al evaluated concurrent gemcitabine with high‐BED (25 Gy/1 fraction, BED_10_ = 87.5 Gy) SBRT for LAPC reported local recurrences in only 19% of patients. They reported 15% grade 2 ulcers and one patient with a duodenal perforation.^20^ Moreover, an early SBRT regimen prior to routine onboard image guidance of 45 Gy/3 fractions (BED_10_ = 112.5 Gy) by Hoyer et al resulted in 5 out of 22 patients developing severe mucositis or perforation.^7^ As a result, modern SBRT regimens use lower doses to maintain safety of therapy. A retrospective series by Moningi et al using dose of 33 Gy in 5 fractions (BED_10_ = 54.8 Gy) showed a median survival from start of radiation of 14 months^21^ while Mahadevan and colleagues used a dose of 24‐36 Gy/2‐3 fractions (BED_10_ range: 52.8‐79.2 Gy) resulting in median survival from diagnosis of 20 months.^4^

More recently, data by Krishnan et al found that high‐BED treatments (BED_10_: 70.4‐100 Gy) using SIB in selected patients lead to improved OS (36% at 2 years) and loco‐regional recurrence free survival, but patients only received dose escalation if their tumors were more than 1 cm away from GI mucosal structures.^11^ This study did not select specific patients for dose escalation but used MRI‐guidance to allow clinicians to make the decision regarding plan adaptation and feasibility based on daily evaluation of internal anatomy. Dose escalation was evaluated with BED_10_ to compare the various radiation treatment regimens. In addition to the survival benefit, high‐dose regimens demonstrated a trend toward significance for improved local control.

The median OS of 10.8 months in our standard‐dose cohort emulated historical controls receiving conventional chemoradiation therapy demonstrating that standard radiation doses may not improve outcomes even if visualization of internal anatomical changes leads to improved localization and motion management. Malik et al reported LAPC patients undergoing chemoradiation range in median survival from 7 to 11 months while the same group in the LAP07 trial reached a median OS of 15.2 months.^1^, ^22^ Of note, the OS outcome in LAP07 was inclusive of the time of induction chemotherapy whereas we calculated OS from start of RT. This accounts for the numerical difference in survival between our standard‐dose cohort and the LAP07 cohort. More impressively, patients in the high‐dose group of this study demonstrated a sizeable survival advantage compared to the standard‐dose group despite this cohort having older age and higher CA 19‐9 levels, which are known negative prognostic factors.^23^ Table 4 lists survival outcomes from previous studies compared to this study cohorts.

**Table 4:** Survival outcomes with RT for inoperable pancreatic cancer

Study	RT technique	Number of patients	Median OS (mo)	2‐y OS (%)
Hammel et al, Phase III^1^	3DCRT	133	15.2	N/A
Krishnan et al^11^, ^a^	Mostly 3DCRT	153	15.0	19
Krishnan et al^11^, ^a^	Mostly IMRT	47	17.8	36
Standard‐dose (current series)^a^	MRI‐guided IMRT and conventional SBRT	20	10.8	30
Huguet et al^9^	IMRT	134	23.0	48
Mahadevan et al^4^	SBRT	39	20.0	N/A
Moningi et al^21^, ^a^	SBRT	88	13.7	15
High‐dose (current series)^a^	Adaptive MRI‐guided Hypofractionated and high‐dose SBRT	24	20.8	49

3DCRT, 3‐D conformal radiation therapy; IMRT, intensity modulated radiation therapy; MRI, magnetic resonance imaging; N/A, not available; OS, overall survival; RT, radiation therapy; SBRT, stereotactic body radiation therapy.

a OS calculated from start of radiation therapy.

FFDF rates were similar between the two groups despite the large difference in OS. We postulate that effective doses of radiation prevent preclinical local failure symptoms such as anorexia, cachexia, and nausea that often preclude effective systemic therapy at progression. In addition, post‐RT pancreatectomy rates did not change with the high‐dose regimens, and the overall rate of resection of 14% was low in this study as compared to historical series.^9^, ^10^ Determining resectability radiographically is difficult after neoadjuvant therapy due to tissue fibrosis complicating imaging interpretation.^24^ Use of high‐dose regimens may increase the risk of fibrosis even further. However, even without the benefit of surgical resection, high‐dose patients had favorable OS outcomes.

Fear of treatment‐related toxicity is a hurdle for using ablative RT doses in pancreatic cancer. Patients in this study experienced an overall acute grade 3+ GI toxicity rate of 7% with one patient experiencing duodenal toxicity. No patient in the high‐dose cohort experienced severe toxicity suggesting that adaptive radiation and decreased use of concurrent chemotherapy diminished this risk. Plan adaptation was frequently used in the high‐dose group to limit dose to OARs when significant internal anatomical changes occurred. Further follow‐up will be required to assess for late toxicities, but it is reassuring that high‐dose adaptive MRgRT appears to be well tolerated acutely.

This study highlights a novel approach of dose escalation for treatment of inoperable pancreatic cancer, but the study design poses limitations which render the findings as hypothesis‐generating. Medically inoperable patients with resectable disease based on imaging criteria were included in this study, but bias from these patients is unlikely given that only two patients met this criterion. Though this was a multi‐institutional study, the sample size was relatively small, and heterogeneity of treatment approaches existed between institutions. We also note that radiographic assessment for tumor response is challenging after delivery of RT and local evaluation of imaging studies can introduce bias. Furthermore, high‐dose RT did not predict for OS on MVA. A larger sample size would be needed to demonstrate significance of high‐dose RT when grouped by BED_10_, yet, it is still important to note that high‐dose RT remained significant on univariate regression and Kaplan‐Meier analyses. Another limitation was that a number of patients had the same prescription dose but heterogeneous delivered dose due to adaptive treatments. Although stratifying patients by BED_10_ of prescription dose did not account for this dose heterogeneity, the swimmer's plot stratified patients by MaxBED_10_to explore dose heterogeneity and its impact on OS. These study data support the evaluation of dose‐escalated regimen in a larger, prospective setting.

## 5 CONCLUSIONS

High‐dose adaptive MRgRT in the treatment of inoperable pancreatic cancer resulted in improved OS in this multi‐institutional study. Adaptive MRgRT provides an innovative technique to administer higher doses of radiation to patients without increasing the risk for acute toxicity. A prospective, phase II multi‐institutional study (NCT03621644) prescribing 50 Gy in 5 fractions (100 Gy BED_10_) using adaptive dose escalation with MRgRT is open, accruing, and will clarify the role of MRgRT for inoperable pancreatic cancer.

## CONFLICT OF INTEREST

The authors listed below report the following financial relationships: NJ reports honoraria from ViewRay, outside the submitted work. LP reports honoraria and stock from ViewRay along with advisory role, speakers' bureau, and research funding from BTG, outside the submitted work. EAM reports travel accommodations from ViewRay, outside the submitted work. AB and FL report honoraria and travel accommodations from ViewRay, outside the submitted work. MCR reports travel expenses from Varian and BTG, outside the submitted work. MFB reports travel accommodations from ViewRay along with research funding from AstraZeneca outside the submitted work, outside the submitted work. PJP reports research funding from ViewRay, outside the submitted work. PL reports honoraria, consulting/advisory role, and travel accommodations from ViewRay, AstraZeneca, and Varian along with research funding from AstraZeneca, outside the submitted work. All other authors have no conflicts to declare.

## AUTHOR CONTRIBUTIONS

Soumon Rudra: data curation, investigation, writing original draft, writing review, and editing. Naomi Jiang: data curation, writing original draft, writing review, and editing. Stephen A. Rosenberg: data curation, writing original draft, writing review, and editing. Jeffrey R. Olsen: investigation, methodology, writing review, and editing. Michael C. Roach: investigation, methodology, writing review, and editing. Leping Wan: formal analysis, writing original draft, writing review, and editing. Lorraine Portelance: data curation, writing review, and editing. Eric A. Mellon: data curation, writing review, and editing. Anna Bruynzeel: data curation, writing review, and editing. Frank Lagerwaard: data curation, writing review, and editing. Michael F. Bassetti: data curation, conceptualization, writing original draft, writing review, and editing. Parag J. Parikh: conceptualization, investigation, methodology, project administration, writing original draft, writing review, and editing. Percy P. Lee: conceptualization, investigation, methodology, project administration, writing original draft, writing review, and editing.
