# Supplementary material for: Using adaptive magnetic resonance image‐guided radiation therapy for treatment of inoperable pancreatic cancer
Source: Cancer Med. 2019 Apr 1;8(5):2123–32. doi: 10.1002/cam4.2100 (PMC6536981; doi:10.1002/cam4.2100)
Supplement: Supplementary file 1 [file CAM4-8-2123-s001.pdf]

Supporting File

Methods: Description of MRI-guided simulation and treatment techniques by institution

Table A1: Dose Constraints for Organs at Risk by Institution

Figure A1: Freedom from Distant Failure from start of Radiation Therapy stratified by Biologically Effective Dose (BED_{10}). Standard error bars displayed at each 6-month timepoint.

Figure A2: Swimmer's Plot of Patient Survival From Date of Diagnosis Stratified by BED_{10} of Maximum Point Dose to Planning Target Volume ($MaxBED_{10}$) [High-dose: $MaxBED_{10}$>90 , Standard-dose: $MaxBED_{10}$≤90]

Methods

Description of MRI-guided simulation and treatment techniques by institution

Site A:

Patients were simulated using a planning CT with oral and IV contrast (unless contrast allergy precluded use) using expiratory breath hold technique. An MRI simulation followed with the same immobilization and breath hold technique. Gross tumor volume (GTV) included the primary tumor and any enlarged regional lymph nodes (greater than 1 cm in short axis) and was delineated using both MRI and CT simulation imaging as well as information from diagnostic imaging. Treatments for patients receiving 18 or more fractions were delivered with respiratory gating. Adaptive treatments were delivered for patients receiving 15 or fewer fractions and required patients to receive a volumetric MRI prior to each fraction. Patients were aligned based on their volumetric MRI and their radiation plan was evaluated on their current anatomy. If the prior plan did not meet target volume coverage or OAR constraints, a new plan was created using the same beam angles. If a new plan that improved coverage and/or constraints could not be generated, then the prior plan was utilized for treatment that fraction.

Site B:

Treatment planning began by obtaining the MRI simulation for each individual patient. Patients were placed head-first supine with a wingboard and arms above their head if tolerated. A 3D volumetric MRI was then obtained at maximum inspiratory breath hold. Next, we assessed the patient's tumor motion, tracking algorithm accuracy, and consistency of the patient's breath hold using Cine MRI scans. A planning CT with a pancreatic protocol and IV contrast was then obtained in the same position at similar breath hold. Treatment was planned for gating (both conventional fractionated and SBRT regimens) with maximum inspiratory breath hold.

The 3-D MRI scan and the CT simulation underwent rigid registration. Tumor was delineated primarily using the contrast enhanced CT imaging locally registered to the MRI which was used as a primary reference scan for contouring normal structures. GTV included primary tumor and grossly enlarged lymph nodes. The elective treatment of lymph nodes (CTV) was at the discretion of the treating radiation oncologist. A 3 mm isotropic expansion was made from GTV or CTV to PTV and used as a respiratory gating boundary. All patients underwent gating during treatment, but there was no daily treatment adaptation at this site.

Site C:

Patients were simulated in the supine position with arms above the head and custom immobilization device to create a reproducible setup. All patients received a planning CT scan with intravenous (IV) contrast (unless precluded due to contrast allergy or baseline creatinine >

1.4 within 6 weeks of simulation) using a Deep Inspiratory Breath Hold (DIBH) technique. A 4-Dimensional (4D) CT planning scan was also obtained as an alternative solution in case the patient could not undergo DIBH technique during treatment. All patients then underwent a 0.35T MRI planning scan with DIBH technique. When available, a recent diagnostic PET-CT image set was registered to the 0.35T MRI planning image set. Contours were delineated using the 0.35T MRI as the primary image set with the other image sets as reference. GTV was defined as the primary tumor and expanded by 3mm to delineate the planning target volume (PTV). No clinical target volume (CTV) was used as no regional lymph nodes were electively irradiated. Stereotactic body radiotherapy (SBRT) was delivered every other day for 2-3 fractions total per week. Image-guidance was performed from a daily acquired volumetric 0.35T MRI obtained prior to each fraction and adaptive treatments were delivered when necessary as determined by the supervising physician. The determination for the necessity of adaptive treatments was done using new deformed contours of the GTV and OARs based on inter-fractional anatomical changes. If the original plan violated any of the OAR constraints, then a new plan was generated by optimizing the original plan utilizing the same beam angles. The new adaptive plan was delivered if this new plan was superior to the original plan with respect to the OAR constraints. For treatment delivery, a DIBH-gated technique was used for all patients using a 3mm gating margin to account for intrafraction tumor motion. Alternatively, if the patient could not undergo DIBH-gated treatments, free-breathing technique using an internal target volume (ITV) approach was performed.

Site D:

Patients were simulated in supine position with one or both arms up with a planning CT and MRI simulation, both during shallow inspiratory breath hold. No oral or I.V. contrast was used for simulation. Patients were instructed not to eat or drink for two hours prior to treatment. Normal and target structures were contoured on the planning MRI with the aid of diagnostic imaging and in cooperation with a dedicated GI radiologist. The GTV included the primary tumor and any immediately adjacent enlarged lymph nodes. A 3 mm margin was added to the GTV to generate the PTV, excluding any overlap with contoured organs at risk (OAR), i.e. the duodenum, stomach, small bowel, liver, colon, spinal cord, kidneys. Treatment was delivered in three fractions per week with prophylactic dexamethasone and ondansetron. After alignment on the GTV, a high-resolution MRI scan in shallow inspiration was repeated prior to each fraction, adjusted where needed in case of small rotations. Deformed OARs were adjusted for the anatomy of the day within the first three cm outside the PTV. The baseline plan was recalculated on the new MRI scan, followed by routine plan adaptation using the same beam angles. All treatments were delivered during repeated shallow inspiration breath holds using an automated gating technique. This breath-hold delivery was facilitated by means of video-feedback of the sagittal tracking image to the patients during delivery.

Site E:

Patients were simulated using a planning CT using inspiratory breath hold technique without oral contrast to obtain a planning study as close as possible to the treatment condition (patients in our institution are instructed to have nothing by mouth for four hours pre-treatment).

IV contrast was not used, but the planning CT was fused with the most recent contrast enhanced diagnostic CT (CECT). An MRI simulation followed with the same immobilization and breath-hold technique. A fusion was performed with available CECT, diagnostic MRI and PET-CT. Normal and target structures were contoured on the planning MRI. GTV included the primary tumor and any adjacent enlarged regional lymph nodes (greater than 1 cm in short axis). A 3 mm margin was added to the GTV to generate the PTV. The following organs at risk (OAR) were also contoured: duodenum, stomach, liver, colon, small bowel, spinal cord, kidneys. The imaging studies and data sets were transferred to the treatment planning system. The target dose was prescribed to the PTV. The original PTV was cropped (PTV mod) away from the duodenum or stomach by 3 to 5 mm to meet the dose constraints when necessary. PTV did not have to be cropped from any other OARs. At the time of plan approval, a tracking volume was created.

Patients were aligned based on their volumetric MRI and their radiation plan was evaluated on their current anatomy. On the MRI of the day, a contouring volume (CV) was generated by adding a 3 cm margin to the PTV. Re-contouring was limited to the GTV and the OARs included within this CV. The original plan was imported on the structure set of the day. If the dose distribution showed that the plan did not meet PTV coverage or OAR constraints, a new plan was generated and delivered to the patient. All treatments were delivered using a breath hold technique. If the GTV was out of the tracking volume by more than 5%, the beam was automatically turned off.

Table A1. Dose Constraints for Organs at Risk by Institution							
**Institution**	**Stomach**	**Duodenum**	**Small Bowel**	**Large Bowel**	**Liver**	**Spinal Cord**	**Kidney**
**Site A**							
25 Fractions	Dmax < 54 Gy<br>V50 Gy < 10 %	Dmax < 60 Gy<br>V55 Gy < 1 cc	Dmax < 54 Gy<br>V50 Gy < 10 %		Mean Dose < 25 Gy	Dmax < 50 Gy	
15 Fractions	Dmax < 45 Gy<br>V50 Gy < 10 %	Dmax < 45 Gy	Dmax < 45 Gy		Mean Dose < 25 Gy	Dmax < 45 Gy	
5 fractions	V33 Gy < 0.5 cc	V33 Gy < 0.5 cc	V33 Gy < 0.5 cc	V33 Gy < 0.5 cc			
**Site B**							
25-28 Fractions	V54 Gy < 0.5 cc	V55 < 0.5 cc	V54 Gy < 0.5cc		Mean Dose < 28-30 Gy	Dmax < 45 Gy	V18 Gy < 30%
5 fractions	Dmax < 25 Gy	Dmax < 25 Gy	Dmax < 25 Gy		Mean Dose < 13 Gy	Dmax < 20 Gy	V15 Gy < 66%
**Site C**							
5 Fractions	V35 Gy < 0.35cc	V35 Gy < 0.35 cc	V20 Gy < 20 cc (Bowel Bag)<br>V35 Gy < 0.35cc		V15 Gy < 1000 cc	Dmax 2.5 Gy / Fraction	Dmax 2.5 Gy / Fraction
**Site D**							
5 Fractions	V36 Gy < 0.1 cc<br>V33 Gy < 1 cc<br>V25 Gy < 20 cc	V36 Gy < 0.1 cc<br>V33 Gy < 1 cc<br>V25 Gy < 20 cc	V36 Gy < 0.1 cc<br>V33 Gy < 1 cc<br>V25 Gy < 20 cc		V12 Gy < 50%		V12 Gy < 25%
**Site E**							
25 Fractions	V55 Gy < 0.5 cc	V54 Gy < 0.5 cc	V54 Gy < 0.5 cc	V54 Gy < 0.5 cc	Mean Dose < 25 Gy	Dmax < 45 Gy	
5 Fractions	V33 Gy < 1 cc	V33 Gy < 1 cc	V33 Gy < 1 cc	V33 Gy < 1 cc	Mean Dose < 20 Gy	Dmax < 20 Gy	

Table A1. Dose Constraints for Organs at Risk by Institution							
**Institution**	**Stomach**	**Duodenum**	**Small Bowel**	**Large Bowel**	**Liver**	**Spinal Cord**	**Kidney**
	V15 Gy < 10 cc	V15 Gy < 10 cc	V15 Gy < 10 cc	V15 Gy < 10 cc	V17 Gy < 700 cc		

Abbreviations: Dmax, maximum dose to organ; VXX Gy, volume of organ receiving XX Gy

Figure A1

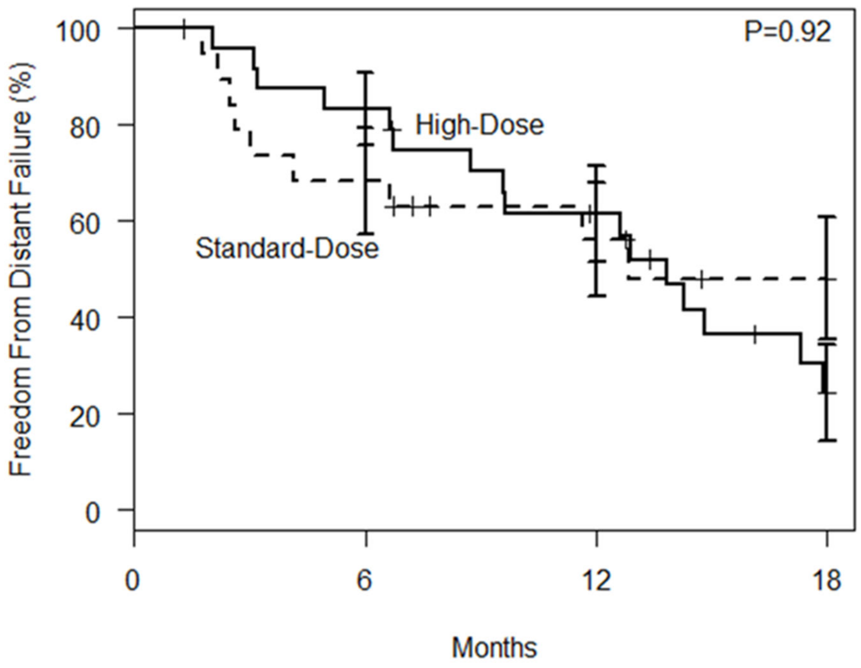

High-Dose	24	20	13	4
Standard-Dose	20	13	8	5

Figure A2

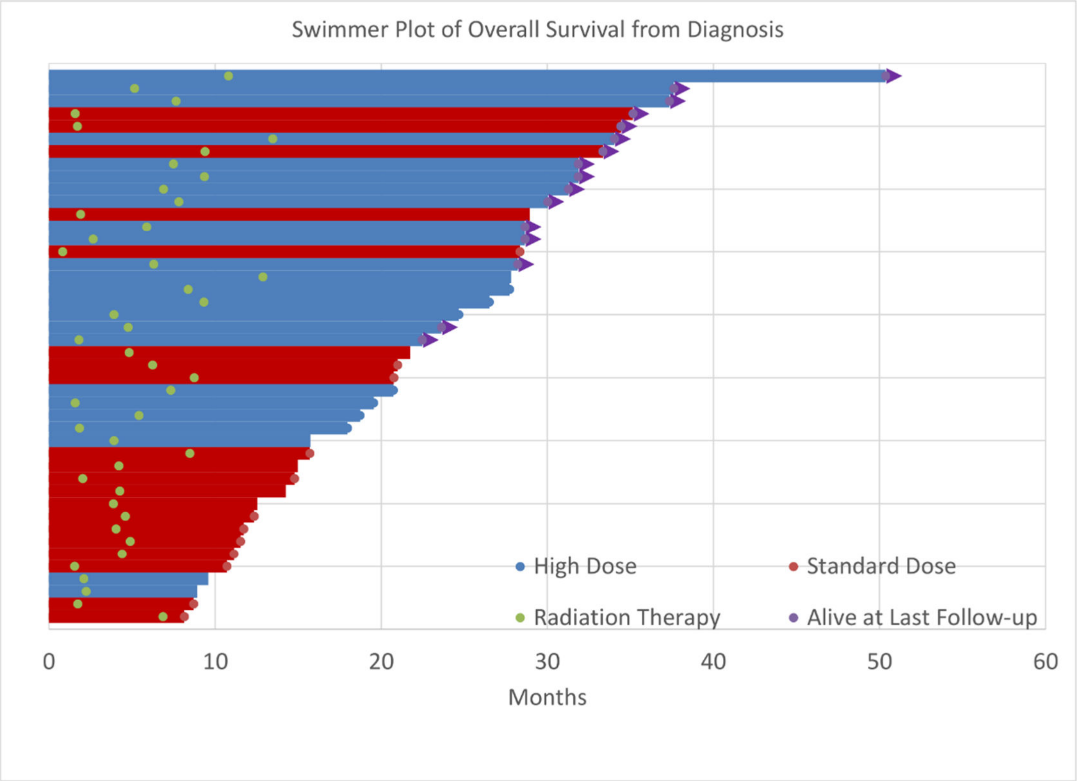

Swimmer Plot of Overall Survival from Diagnosis
High Dose
Standard Dose
Radiation Therapy
Alive at Last Follow-up
0
10
20
30
40
50
60
Months
